# Nurses' knowledge and practices of physical restraints in intensive care units: An observational study

**DOI:** 10.1002/nop2.625

**Published:** 2020-09-14

**Authors:** Maysa H. Almomani, Wejdan A. Khater, Baha'a Aldin Abdel‐Latif Qasem, Rachel A. Joseph

**Affiliations:** ^1^ Department of Adult Health Nursing Faculty of Nursing Jordan University of Science and Technology Irbid Jordan; ^2^ Speciality Hospital Amman Jordan; ^3^ Department of Nursing College of Health Sciences West Chester University of Pennsylvania West Chester PA USA

**Keywords:** intensive care units, nurses' knowledge, physical restraints, practice

## Abstract

**Aim:**

To investigate the knowledge and practice of physical restraints (PR) among Jordanian intensive care unit (ICU) nurses.

**Design:**

A descriptive, observational design was used.

**Methods:**

A convenience sampling was used to recruit participants. We examined the knowledge of PR in 301 nurses (knowledge check) and the real‐time practice of PR in 81 nurses (direct observation) in ICU. A knowledge questionnaire was used to collect data on knowledge about PR use, and data on their practice of PR were observed and documented using an observation checklist.

**Results:**

The mean scores of nurses' knowledge and practices were 61.5 (*SD* = 12.1) and 57.4 (*SD* = 9.7), respectively. More than half of nurses had poor knowledge of PR use and incorrect practice of implementing PR (51.5% and 60.5%, respectively). Results indicated a positive correlation between nurses' knowledge and their use of PR.

## 1 INTRODUCTION

Physical restraints (PR) are defined as "…any action or procedure that prevents a person's free body movement to a position of choice and/or normal access to his/her body by the use of any method, attached or adjacent to a person's body that he/she cannot control or remove easily." (Bleijlevens, Wagner, Capezuti, & Hamers, 2016, p. 3). Physical restraints are widely used, particularly in the care of critically ill patients, to ensure their safety and to protect them from fall, injury and/or unintended harm (Birgili & İzan, 2019; Kandeel & Attia, 2013; Pellfolk, Gustafson, Bucht, & Karlsson, 2010; Wang, Zhu, Zeng, & Xiong, 2019). However, applying PR is often considered unsafe and unacceptable (Luk, Burry, Rezaie, Mehta, & Rose, 2015; Martin & Mathisen, 2005); therefore, accreditation standards, guidelines and legislation recommend minimization of PR use (Azab & Negm, 2013; Luk et al., 2015; Möhler & Meyer, 2014; Taha & Ali, 2013). However, in some situations, it can become a necessity for the safety of the patient and caregivers.

Optimal use of PR may reduce harm to the patient (by limiting undesirable movements) and caregivers. For example, restraints are used to prevent patients' injuries and falls, control agitated patients, control disruptive behaviours and prevent patients from removing tubes or medical equipment connected to their body (Nasrate, Shamlawi, & Darawad, 2017). However, its application may be unsafe and associated with adverse outcomes, such as an increase in nosocomial infections, length of hospital stays, physical and emotional trauma and even death (Birgili & İzan, 2019; Demir, 2007; Evans, Wood, & Lambert, 2003; Kwok et al., 2012). Thus, the use of PR can exacerbate economic burdens on the family, healthcare organization and government. Furthermore, the ethical dilemma related to PR usage has negative consequences on the nursing staff (Chuang & Huang, 2007).

Nurses in intensive care units (ICUs), who try to manage the patient and the technology used for care, are the key decision‐makers regarding the application of PR (Lane & Harrington, 2011; Luk et al., 2015; Minnick, Mion, Johnson, Catrambone, & Leipzig, 2007; Pellfolk et al., 2010). Many factors influence their decisions which can be grouped into three categories: patient factors, nurse factors and environmental factors (Azab & Negm, 2013; Lane & Harrington, 2011; Pellfolk et al., 2010). Factors related to patients include patient's age, medical diagnosis and the presence of invasive devices (Al‐Khaled, Zahran, & El‐Soussi, 2011; Lane & Harrington, 2011; Martin & Mathisen, 2005; Pellfolk et al., 2010). Factors related to nurses include nurse's age, years of experience, nurse's attitude, knowledge and qualifications (Al‐Khaled et al., 2011; Azab & Negm, 2013; Martin & Mathisen, 2005; Pellfolk et al., 2010). Environmental factors in ICU include those which would increase patient agitation, anxiety and deliria such as noise from alarms of equipment, other patients or the healthcare personnel, lighting and presence of multiple caregivers (Bray et al., 2004).

While the use of PR is at times necessary in ICUs, it is a debatable practice (Birgili & İzan, 2019; Wang et al., 2019) which gained attention from government, healthcare organizations, accrediting agencies, lawyers and researchers in recent years. The use of PR is one of the quality indicators of healthcare institutions (Spilsbury, Hewitt, Stirk, & Bowman, 2011). Nurses have an active role in decision‐making about PR, and they may have negative feelings, such as frustration and guilt, regarding its use (Al‐Khaled et al., 2011; Möhler & Meyer, 2014). PR use can create a barrier between the nurse and the patient and the family, thereby making their relationships ineffective and less therapeutic (Duxbury, 2002). Questions about the benefits of PR use emerge along with associated practical, ethical and legal issues (Martin & Mathisen, 2005). Nurses' knowledge and practices are important factors that influence optimal patient care (Christensen, 2011). Therefore, it is important to examine the knowledge about PR and their real practice.

## 2 BACKGROUND

Literature indicates that nurses' knowledge about PR is less than optimal (Azab & Negm, 2013; El‐sol & Mohmmed, 2018; Eskandari, Abdullah, Zainal, & Wong, 2017; Kassew, Dejen Tilahun, & Liyew, 2020; Pradhan, Lama, Mandal, & Shrestha, 2019; Suliman, Aloush, & Al‐Awamreh, 2017; Taha & Ali, 2013). A knowledge deficit may have an impact on decision‐making regarding PR, thus decreasing the nurses' ability to provide optimum care (Yeh et al., 2004). Additionally, potentially serious complications can occur in patients who are physically restrained (Lane & Harrington, 2011; Suliman, 2018; Taha & Ali, 2013). Möhler and Meyer (2014) emphasized that understanding nurses' knowledge about PR use is essential to address the problem related to that. A satisfactory level of nurses' knowledge of PR is found to be associated with their practice of PR and related complications or events (Azab & Negm, 2013; Heeren et al., 2014; Taha & Ali, 2013).

Many hospitals in developing countries do not have standardized guidelines or clear policies for the use of PR (Taha & Ali, 2013). In Jordan, a lack of such guidelines and policies may lead to improper and unsafe practice, thus affecting the quality of care (Suliman, 2018). Training and developing practice guidelines would improve nurses' knowledge and help implement safe practice (Taha & Ali, 2013). Before such steps are taken, it is essential to understand what the current knowledge and practice of nurses regarding PR use are.

There is an increased concern about patient safety related to the overuse of PR in Jordan. Suliman (2018) found that PR use is a common practice in Jordan. The prevalence of restraint use in ICUs in five public hospitals and one university‐affiliated hospital in Jordan was 35.8%, and this includes chemical restraints. The prevalence rate varied between units and the highest rate was in the surgical ICUs (57.1%). Also, restrained patients were observed to have physical complications, such as bruising, redness and oedema. Lack of policies and regulations for PR use in Jordanian hospitals means that PR is used more frequently and associated with significant complications or even death.

There is a lack of studies exploring the use of PR in Jordan. Lack of adequate studies on the practice of PR and the nurses' knowledge in the Jordanian context leaves the health care system with no evidence for practice. Suliman et al. (2017) reported insufficient knowledge, negative attitudes and unsafe practices of PR use among ICU nurses from three public hospitals and one university‐affiliated hospital. Suliman (2018) and Suliman et al. (2017) reported that nurses in Jordan often did not document PR and initiate restraint without obtaining a physician's decision because they thought that PR is required for patient safety and protection (Suliman, 2018; Suliman et al., 2017). Nasrate et al. (2017) reported that more than half of ICU nurses in Jordan did not know if their hospital had a PR policy; yet, most used PR frequently. However, self‐administered questionnaires to assess nurses' practice of PR could under‐reveal the actual practice in Jordanian hospitals. The observational method was recommended to validate the findings (Nasrate et al., 2017; Suliman et al., 2017). Thus, to fill this gap in the literature, this study used real‐time observations as a method to assess nurses' practices along while their knowledge on PR use was assessed using a self‐reported questionnaire.

Hitherto, studies that observed the PR‐related practice of nurses are limited in the literature. Therefore, the purpose of this study was to examine the nurses' knowledge and observe their practice of PR use in adult ICUs in Jordan. In addition, we explored the relationship between their levels of knowledge and their practice of PR and the impact of socio‐demographic characteristics on the knowledge and practices of PR.

### 2.1 Research question

The research questions included the following:

1. What is the level of nurses' knowledge of PR in ICUs in Jordan?
2. What are the nurses' practices of PR use in ICUs in Jordan?
3. What is the relationship between the level of nurses' knowledge and practices of PR in ICUs in Jordan?
4. Are there differences between nurses' socio‐demographic characteristics (age, gender, years of experience in ICUs, education and type of health system) in their levels of knowledge and practice of PR?

## 3 METHODS

### 3.1 Design

A descriptive, correlational and observational design was used to examine different aspects of nurses' knowledge and to observe their practices of PR use in ICUs in Jordan.

### 3.2 Participants and settings

The study was conducted in nine ICUs located in the three largest governorates in Jordan. There are only nine ICUs in these governorates. Criteria for participation include the following: nurses currently working in ICU have at least 1 year of experience in ICU and fluency in Arabic. Nurses with a diploma and baccalaureate degree could participate as all of them provide direct patient care. Registered Nurses, who were chosen and trained as observers in each ICU, were excluded from participation.

A convenience sample of 301 nurses who met the criteria and consented was recruited and assessed for the knowledge using a self‐reported questionnaire. Based on Cohen tables, the overall required sample size was 85 from all hospitals (Cohen, 1992), with a power of 0.80, an alpha level of 0.05 and a medium effect. Maximum available nurses were recruited for the self‐report of knowledge; however, only 81 of them were observed for their practice of application of PR. This was four less than the required sample size.

### 3.3 Data collection tools

Two measures were used for data collection; an instrument to assess nurses' knowledge regarding PR use in ICUs and a checklist for observation of their practices of PR use. The instrument to measure nurses' knowledge of PR, developed originally in the Arabic language by Taha and Ali (2013), consisted of two parts: first, a demographic section that consisted of age, gender, number of years of clinical experience in ICUs, type of healthcare service (Government, private and University Hospitals), qualifications (diploma or degree type) and two questions related to the awareness of PR policies in the hospital and the perceived awareness of PR use; and second, 35‐item multiple‐choice questions to assess nurses' knowledge regarding PR such as definitions, indications, types, alternatives, procedures, precautions, contraindications, complications and barriers to use it. The responses were evaluated using a model answer key provided by Taha and Ali (2013). Each correct or satisfactory response gets a score of 1, and an incorrect or unsatisfactory response carries a score of zero. The maximum possible score was 35, and the individual scores were converted to a percentage. A score of 60% or above is considered satisfactory knowledge (Taha & Ali, 2013).

For the observation component of the study, a checklist developed by Taha and Ali (2013) was used to assess the practice of PR. This also has two parts: first, general information such as observation site, observation time and nurse to patient ratio and the second, a 19‐item checklist that covers the PR practices, including the assessment, preparation, application, postcare (after application), maintenance and documentation. The observed practices were compared with the standardized procedures available from the Lippincott manual. Accordingly, each item was given a score of 1 for complete and correct practice and a score of 0 for incomplete, incorrect and missed step in practice. The maximum possible score is 19, and the actual score of the nurses was converted to percentages. A score of 60% or above was considered acceptable practice (Taha & Ali, 2013).

Literature supports adopting different levels for knowledge and practice scores (Karlsson, Bucht, Eriksson, & Sandman, 2001; Lane & Harrington, 2011): a score above 80% was considered good, 60%–80%, moderate and less than 60%, poor. The reliability of the questionnaire was assessed in different studies and ranged from 0.74–0.78 (Elshamy, Sharif, & Shebl, 2014; Taha & Ali, 2013). In the current study, the Cronbach's alpha was 0.77 (some items deleted because of zero variance; in pilot = 0.88).

### 3.4 Validity and inter‐observer reliability

Content and face validity of the instruments though established in a previous study in Egypt (Taha & Ali, 2013) were re‐examined for use in the Jordanian context. A panel of seven experts in the nursing and medical fields (four members of the professional nursing staff and three members of the medical staff) evaluated the tools for content and was modified based on their recommendation by rewording some items for clarity. The content validity index was 1.0.

Eight data collectors were trained to observe the nurses while applying PR, based on a checklist. The data collectors watched a video recording describing the observation, and they were all given the same training sessions by researchers with detailed guidelines about whom, what, when and for how long to observe. To decrease the Hawthorne effect, nurses were generally informed about the expected data collection and upcoming observations, but not about the time and date and who would collect data and observe their practice. The observers did not complete the checklist while the PR application is being performed but completed it immediately after the procedure. Inter‐observer reliability was assessed by making two observers score a nurse applying PR, as shown in the video. The intra‐class correlation coefficient was .82, which indicated an excellent inter‐observer agreement (Cicchetti, 1994).

### 3.5 Data collection procedures

Names of nurses who were working in the selected ICUs at the time of the study were obtained from the heads of the units after obtaining IRB approvals. They were approached and given information about the study, and those who consented to participate completed the self‐reported knowledge questionnaire. Those nurses who had to apply PR during their shift were observed, and a checklist was completed by the trained observers immediately after the procedure. After this, they were approached by the researcher to request permission to use the data from the PR observation checklist and consenting nurses signed the second consent form. The consent was signed after the event to avoid anxiety and feelings of discomfort on knowing that that they are being observed. Feelings of being watched itself can make them self‐conscious and can produce errors in procedures. All hard copy data were stored in a locked cabinet in the principal investigator's office and analysed later.

### 3.6 Observations

Trained data collectors who were Registered Nurses working different shifts in the units, observed nurses' practices of PR over 16 weeks. One nurse was observed only once by two observers; however, all the PR applications seen during the data collection period were recorded without repetition. The observation was based on the competency checklist and completed immediately after the PR application. The nurse observee was by self‐selection because their patient required PR during that shift. The relationship between the observee and the observer was not specified; we assumed they have a regular collegial relationship.

### 3.7 Ethics

Institutional review boards at Jordan University of Science and Technology (IRB #13/79/2014) and the Ministry of Health (IRB #15445) approved the study. Additionally, permissions were obtained from the selected hospital's administrative authorities and heads of the selected ICUs. Those who consented to participate signed consent forms twice: once prior to the completion of the knowledge tool and the second after observing their practice allowing us to use the observational data for research. Participation was voluntary, and privacy and confidentiality were assured. They were assured that the observation is for the practice of applying PR, not the nurses themselves.

Accompanying each questionnaire was a cover letter describing the objectives of the study and a statement that the participation was voluntary. The participants were assured that the collected data would be used solely for the study's objectives and participants were informed of their right to refuse participation, or their ability to withdraw at any time, without penalty.

### 3.8 Analysis

Descriptive statistics (frequencies, percentages, mean and standard deviation) were used to describe the study's sample and the responses to the items on knowledge and practices regarding PR use. A Pearson correlation test was used to test the association between total knowledge scores, total practice scores, years of experience and age of nurses. A *t* test and ANOVA were used to compare the knowledge and practice scores between groups of nurses according to demographic characteristics (sex, education level and health sectors). Statistical significance was set at a *p‐*value <.05.

## 4 RESULTS

### 4.1 Nurses' socio‐demographic characteristics

A total of 301 nurses participated in the study and 81 nurses were observed for their practice of applying PR. Most of the nurses were young (mean = 27.84; *SD* = 2.7), held a bachelor's degree (88.7%) and had 3 years or less of ICU experience (73.8%). Table 1 describes the nurses' socio‐demographic characteristics.

**Table 1:** Socio‐demographic characteristics of participant nurses (*N* = 301)

Variable	Mean	*SD*	*N* (%)
Age	27.84	2.75	
Gender			
Male			163 (54.2%)
Female			138 (45.8%)
Marital status			
Single			151 (50.2%)
Married			150 (49.8%)
Educational background			
Diploma			21 (7.0%)
Bachelor			267 (88.7%)
Master			13 (4.3%)
Years of experience in ICU	2.74	1.77	
1–3 years			222 (73.8%)
4–6 years			64 (21.2%)
7–9 years			15 (5.0%)
Working place			
Governmental hospital			139 (46.2%)
Private hospital			72 (23.9%)
University hospital			90 (29.9%)
Considering themselves as having enough knowledge			
Yes			158 (52.5%)
No			143 (47.5%)
Awareness of physical restraint policies in hospital			
Yes			120 (39.9%)
No			181 (60.1%)

### 4.2 Level of knowledge of PR use in Jordanians ICU nurses

The mean scores of nurses' knowledge regarding the use of PR were 61.5 (*SD* = 12.1). Based on their response to the question: “Do you have adequate knowledge about PR use?”, 52.5% of nurses perceived they possess adequate knowledge. Yet, the total knowledge scores indicated that 51.5% of nurses had poor (unsatisfactory) knowledge. Only 12% had good knowledge, while 36.5% had moderate knowledge (Table 2).

**Table 2:** Items of the knowledge questionnaire about use of physical restraint (*N* = 301)

Knowledge item	Satisfied	Unsatisfied
	*N* (%)	*N* (%)
Definition of physical restraint	298 (99%)	3 (1%)
Decision of restrain order	190 (63.1%)	111 (36.9%)
Indications of physical restraint	130 (43.2%)	171 (56.8%)
Physical restraint considers as optimal solution	116 (38.5%)	185 (61.5%)
Physical restraint sites	233 (77.4%)	68 (22.6%)
Physical restraint instruments	42 (14%)	259 (86%)
Physical restraint contraindications	162 (53.8%)	139 (46.2%)
Doing exercise for restrained patient	289 (96%)	12 (4%)
Causes of doing exercise for restrained patient	154 (51.2%)	147 (48.8%)
Release of physical restraint	293 (97.3%)	8 (2.7%)
Causes of releasing physical restraint	159 (52.8%)	142 (47.2%)
Presence of direct complications for physical restraint	300 (99.7%)	1 (0.3%)
Types of direct complications for physical restraint	38 (12.6%)	263 (87.4%)
Presence of indirect complications for physical restraint	223 (74.1%)	78 (25.9%)
Types of indirect complications for physical restraint	28 (9.3%)	273 (90.7%)
Presence of psychological complications for physical restraint	268 (89%)	33 (11%)
Types of psychological complications for physical restraint	71 (23.6%)	230 (76.4%)
Types of potential complications for physical restraint	19 (6.3%)	282 (93.7)
Presence of alternatives for physical restraint	202 (67.1%)	99 (32.9%)
Types of alternatives for physical restraint	45 (15%)	256 (85%)
Barriers for physical restraint use related to environment	81 (26.9%)	220 (73.1%)
Barriers for physical restraint use related to nurses	98 (32.6%)	203 (67.4%)
Barriers for physical restraint use related to patients	127 (42.2%)	147 (57.8)
Physical restraint considers as a part of treatment	301 (100%)	0 (0%)
Importance of doing exercise for restrained patient	294 (97.7%)	7 (2.3%)
Documentation of physical restraint	188 (62.5%)	113 (37.5%)
Time period for release physical restraint	74 (24.6%)	227 (75.4%)
Written doctor order for physical restraint every 24hrs	127 (42.2%)	174 (57.8%)
The necessity of observation restrained patient every 15 min	130 (43.2%)	171 (56.8%)
Doing airway examination for physically restrained patient	268 (89%)	33 (11%)
Decreasing physical restraint if agitation decreases	280 (93%)	21 (7%)
The necessity of assessing extremities for neurovascular function	301 (100%)	0 (0%)
Total knowledge score		
Mean (*SD*)		61.5 (12.1)
Range		34.3–91.4
Levels of knowledge		*N* (%)
Poor knowledge (<60%)		155 (51.5%)
Moderate knowledge (60−80%)		110 (36.5%)
Good knowledge (>80%)		36 (12.0%)

All nurses considered the application of PR as part of the patients' treatment plan and knew the need for neurovascular assessment of the restrained extremities. Most had satisfactory answers regarding the presence of direct complications (99.7%) and the importance of passive exercise for the extremities (97.7%). A detailed report of the answers is given in Table 2.

However, nurses had unsatisfactory knowledge of the indications (56.8%) and contraindications (46.2%) for applying restraints. About 61.5% of nurses considered applying PR as an optimal solution to deal with agitated patients. Most nurses did not know the PR instruments (86%), types of alternatives for restraints (85%) and types of potential (93.7%), indirect (90.7%) and direct (87.4%) complications. The frequencies and percentages of satisfied (correct) and unsatisfied (incorrect) answers to each of the 35 questions are listed in Table 2.

### 4.3 Practices of PR in Jordanian ICUs

As shown in Table 3, the overall practice of applying PR was unsatisfactory (mean = 57.4, *SD* = 9.7). Only 2.5% of nurses whose practice was observed demonstrated good practice, while 37.0% had moderately acceptable practice. A large proportion (60.5%) of nurses had poor or unacceptable practices. The major area of concern is in their preparation, post‐PR care and maintenance of PR. In assessment; they failed to identify the patients (check the ID bracelet) (92.6%) or introduce themselves (87.7%). Yet, most nurses provided privacy for patients (90.1%) and assessed patients for comfortable positions and good alignment (90.1%).

**Table 3:** Items of the observation checklist for nursing practice of physical restraints (*N* = 81)

Practice item	Done correctly & completely	Done incorrectly, incompletely, or not done
	*N* (%)	*N* (%)
Assessment and preparation phase		
1‐ Identify patient. Check the ID bracelet for patient	6 (7.4%)	75 (92.6%)
2‐ Introduce yourself to the patient.	10 (12.3%)	71 (87.7%)
3‐ Explain procedure to the patient before beginning.	38 (46.9%)	43 (53.1%)
4‐ Practice hand washing.	66 (81.5%)	15 (18.5%)
5‐ Provide privacy for the patient.	73 (90.1%)	8 (9.9%)
6‐ Make sure patient is comfortable and in good alignment.	73 (90.1%)	8 (9.9%)
Application phase		
7‐ Put the bed rails on the bed	50 (61.7%)	31 (38.3%)
8‐ Pad bony areas. Follow nurses' instruction and the care plan.	63 (77.8%)	18 (22.2%)
9‐ Secure the restraint so it is snug but not tight.	67 (82.7%)	14 (17.3%)
10‐ Adjust the straps if the restraint is too loose or too tight	57 (70.4%)	24 (29.6%)
11‐ Tie the straps to the non‐movable part of the bed.	75 (92.6%)	6 (7.4%)
Post care and maintenance phase		
12‐ Check on the patient at least every 15 to 30 min	2 (2.5%)	79 (97.5%)
13‐ Observe neurovascular function and patient's position and needs	2 (2.5%)	79 (97.5%)
14‐ At least every 2 hr, release the tie, change the patient's position. provide passive range of motion exercise and assess the condition of the skin under the device.	35 (43.2%)	46 (56.8%)
15‐ Provide access to a call bell or other method to summon the nurse.	50 (61.7%)	31 (38.3%)
16‐ Assess for the continued need for the restraint.	70 (86.4%)	11 (13.6%)
Documentation phase		
17‐ Ongoing documentation related to physical restraint use	9 (11.1%)	72 (88.9%)
Total practice score		
*M* (*SD*)		57.4 (9.7)
Range		38–81
Level of practice		*N* (%)
Poor practice (<60%)		49 (60.5%)
Moderate practice (60−80%)		30 (37.0%)
Good practice (>80%)		2 (2.5%)

In post‐PR care and the maintenance phase, 97.5% of nurses failed to check patients every 15 to 30 min or observe their neurovascular functions. Moreover, only 43.2% of nurses released the tie, changed the patients' position and provided a passive range of motion exercise. Most nurses (88.9%) applied PR without a written order by a provider and did not document. However, most nurses had completed the correct practice of the physical application of Restraints itself. Table 3 describes nursing practices regarding PR use, according to each item in the observation checklist.

### 4.4 The relationship between the nurses' level of knowledge and their practice of PR use

A positive correlation between the scores of nurses' knowledge and practice (obtained by observation) was identified, *r* = .93, *p* < .001. This indicates nurses with higher knowledge scores have better practice scores.

### 4.5 Socio‐demographic variables and levels of nurses' knowledge and practices

The nurses' knowledge score had a positive correlation with the number of years of experience in the ICU, *r* = .73, (*p* < .001) and nurses' age, *r* = .54, (*p* < .001). Similarly, the total practice score had a positive correlation with their number of years of experience in the ICU, *r* = .62, (*p < *.001) and their age, *r* = .38, *(p* < .001). This indicates that the overall knowledge and practice scores increase with the number of years of experience in the ICU and with their age. Yet, females and males did not differ significantly in their total knowledge scores, *t* (299) = 0.49, (*p* > .05) or practice scores, *t* (79) = 1.26, (*p* > .05).

Also, total knowledge scores were significantly different between groups of various educational backgrounds, *F* (2, 298) = 13.9, *p* < .001. Nurses with master's degrees obtained significantly higher total knowledge scores than those with bachelor's degrees and/or diplomas. The difference between these groups in their total practice scores was not significant, *F* (2, 78) = 2.09, *p* > .05. This might be because the number of nurses who were observed for their procedural correctness was less than those who were assessed for their knowledge.

Furthermore, there was a significant difference in the total practice scores between the nurses from different types of healthcare sectors, *F* (2, 78) = 3.97, *p* < .05. Nurses in university‐affiliated hospitals obtained significantly higher total practice scores than those in private and governmental hospitals. However, there was no significant difference in the total knowledge scores between the nurses from different health care sectors, *F* (2, 298) = 0.12, *p* > .05.

Further analysis, using multiple linear regression, was conducted to explore whether the relationship between nurses' knowledge and practice was significant after controlling the demographic variables. The regression model explained 89.9% of the variance and that the model was a significant predictor of nurses' practice of PR use, *F* (8, 72) = 80.01, *p* < .001. Nurses' knowledge was a significant predictor of their practices (B = 0.998, *p* < .001), and nurses' practice of PR use was increased by 0.998 unit (95% CI = 0.36, 0.45) as their knowledge score higher by a unit.

## 5 DISCUSSION

This study examined the knowledge and practice of PR use among 301 ICU nurses. Although the results of the study had indicated that more than half of the nurses perceived that they had enough knowledge related to PR, most nurses, unfortunately, had unacceptable practice regarding the use of PR. These findings are congruent with the findings of several studies that nurses lack knowledge regarding PR use and have a low level of practice as well (Al‐Khaled et al., 2011; Fariña‐López et al., 2014; Taha & Ali, 2013). There is incongruence between their perception and real knowledge of PR. This finding may be due to their everyday experiences of using PR rather than evidence‐based (Hantikainen & Kappeli, 2000).

We can argue nurses' knowledge reflects their previous education and experiences. Thus, a lack of knowledge could be due to the absence of in‐service training or special emphasis on appropriate PR use both in graduate and undergraduate curricula and lack of clearly written policies and procedures regarding PR in the different hospitals (Azab & Negm, 2013; Cannon, Sprivulis, & Mccarthy, 2001; Nasrate et al., 2017; Suliman et al., 2017; Taha & Ali, 2013). Few nurses in Jordan had received previous in‐service education or training regarding PR use (Nasrate et al., 2017; Suliman et al., 2017). In the current study, only 4% reported having previous knowledge about PR through special training. Similarly, it was reported that only 35.7% of nurses in Jordan had received previous PR education (Suliman et al., 2017) and 42.5% had received previous PR training (Nasrate et al., 2017). Furthermore, lack of nurses' knowledge and practices could be explained by a lack of nursing experiences in ICU, because nurses' experience enhances their daily practices and performance (Al‐Khaled et al., 2011). For instance, most nurses in the current study have 3 years or less of nursing experience in the ICU, which may have contributed to such findings.

In this study, most PR applications were directed by nurses without to physician' orders, which may expose nurses to legal issues, if or when they incorrectly apply PR (Choi & Song, 2003; De Jonghe et al., 2013). Similarly, previous studies found nurses initiated PR and released it without physician orders (Azab & Negm, 2013; Choi & Song, 2003; Demir, 2007; Eşer, Khorshid, & Hakverdioğlu, 2007; De Jonghe et al., 2013; Kandeel & Attia, 2013; Suliman, 2018; Suliman et al., 2017)^.^ However, evidence showed that nurses require physician orders to apply PR and this order should be renewed by the physician at varying frequencies based on the type of restraint; for example, every 24 hr and the date and time must be included (Maccioli et al., 2003). Furthermore, one‐third of nurses believed that the nursing staff should be responsible for deciding on the initiation of PR (Azab & Negm, 2013). This might be related to the fact that nurses perceive themselves as being responsible for restraining their patients since they spend more time than physicians at the bedside (Cho et al., 2006; Eşer et al., 2007). Further, it was found that PR is also used to restrict the movement of confused and agitated patients especially when there is a shortage of nursing staff (Perez, Peters, Wilkes, & Murphy, 2019; Suliman et al., 2017).

Although special devices and supplies are recommended to physically restrain patients, consistent with previous studies (Eşer et al., 2007; Kandeel & Attia, 2013)_,_ nurses in our study used gauze pads and rolls to restrain patients, due to lack of knowledge, lack of availability of alternate measures and heavy workload (Choi & Song, 2003; Demir, 2007; Eşer et al., 2007; Kong & Evans, 2012; Minnick et al., 2007; Saarnio & Isola, 2010). Inappropriate use of PR has adverse physiological and psychological effects (Demir, 2007; Eşer et al., 2007; Martin & Mathisen, 2005). More specifically, using inappropriate materials for restraining patients physically, such as gauze rolls, may cause impaired circulation, pressure sores and skin complications, thus compromising patient safety (Azab & Negm, 2013; Evans et al., 2003; Kandeel & Attia, 2013).

The application of PR may violate the principles of respect for the dignity and autonomy of others (Gastmans & Milisen, 2006). Unfortunately, approximately more than half of nurses who used PR in the current study did not explain policies and procedures regarding PR to the patients or their families, or why the restraints were being applied; most nurses did not introduce themselves to the patients and did not verify the identity of patients before applying PR. Azab and Negm (2013) reported similar findings where nurses lacked knowledge of patients' rights and were unaware of ethical and legal issues regarding PR use. This shows the need to enhance the awareness of ethical and legal issues related to PR use and patients' rights and enhance the quality of care. A lack of explanation regarding the use of PR may increase their anxiety and agitation (Cheung & Yam, 2005; Gastmans & Milisen, 2006).

Physically restrained patients should be assessed every 15–30 min, and the assessment should focus on the neurovascular status of the extremities (Agens, 2010; Kandeel & Attia, 2013; Maccioli et al., 2003). However, almost all nurses in this study did not assess patients every 15–30 min, completely, correctly, or missed it altogether. Furthermore, more than half of the nurses did not know the necessity of this frequent check. Lack of knowledge regarding ongoing assessments for physically restrained patients and lack of training regarding the application of PR may contribute to such practices.

Although the American College of Critical Care Medicine Task Force 2001–2002 emphasized the importance of conducting physical exercises for restrained extremities (Maccioli et al., 2003), most of the nurses in this study confirmed their adherence to this recommendation every 2–4 hr; though, less than half of the observed nurses actually released the tie, changed patients' position and provided passive ranges of motion exercise. Observation of nurses' practices gave real‐time information about the practice which is more valid than self‐reporting.

Most nurses in our study did not document their interventions. Only nine out of the 81 nurses in the observation group documented, which were mostly during the evening/B shift; they also had written medical orders and family members were present. These findings agree with that of other studies that nurses rarely documented the application of PR (Azab & Negm, 2013; Choi & Song, 2003; Kandeel & Attia, 2013; Morrison et al., 2000). A possible argument can be, nurses believe that the application of PR is unacceptable and unethical (Gallagher, 2011; Martin & Mathisen, 2005). Furthermore, nurses may consider PR application as an insignificant aspect of the practice (Al‐Khaled et al., 2011).

From our observations, we noticed that most PR applications occur in the night shifts especially after midnight; previous studies reported similar findings— most PR occurs during the night shift (Al‐Khaled et al., 2011; De Jonghe et al., 2013; Martin & Mathisen, 2005). A lower number of nurses during night shift compared with the heavy workload in night shifts may explain the higher use of PR to manage patients and compensate for the shortage of the nurses (Al‐Khaled et al., 2011; Azab & Negm, 2013; De Jonghe et al., 2013; Lai, 2007; Suen et al., 2006). The absence of patients' families may increase PR use in the night shifts (Al‐Khaled et al., 2011) because patients might become calmer in the presence of their families (Al‐Khaled et al., 2011; Fitzgerald, Carroll, Elliott, & Gonzalez, 2004). Organizational issues, such as the absence of supervision, during the night shift also may increase the number of PR during night shifts (Lee et al., 2003).

We found that nurses' level of knowledge regarding the application of PR was strongly associated with their levels of practice. Similar findings were reported by others (Al‐Khaled et al., 2011; Azab & Negm, 2013; Fitzgerald et al., 2004; Karlsson et al., 2001; Taha & Ali, 2013) who found a significant relationship between nurses' knowledge and practice regarding PR.

In this study, knowledge and practice levels are positively correlated with the number of years of experience in ICU and their age. Nurses with more experience in the ICU applied PR efficiently than other nurses, which can be explained by the nurses' daily duties, which improve their performance (Tilly & Reed, 2006). Similarly, Al‐Khaled et al. (2011) found a significant relationship between nurses' practice and both their ICU experience and age. However, two previous studies did not find any relationship between nurses' knowledge and practice with both of their ICU experience or their age (Azab & Negm, 2013; Taha & Ali, 2013).

As for years of experience, nurses' level of education was found to have a significant relationship with the total knowledge scores. More specifically, nurses with master's degrees obtained significantly higher total knowledge scores, than those with a bachelor's degree and/or diploma. This might be due to the fact nurses with a master's degree may spend more time learning, than those with bachelor's degrees and/or diplomas. However, Azab and Negm (2013) found that the educational background of nurses did not affect their knowledge regarding PR use. Nurses in this study have different educational backgrounds than those who participated in Azab and Negm study.

While the educational backgrounds of nurses were found to be significant in relation to their knowledge, we did not find any significant difference in the total practice scores between groups of nurses. Similarly, Al‐Khaled et al. (2011) found that nurses with higher qualifications have better practice than others. Different educational programmes do not highlight specific topics such as PR. Further, some reports suggest that Jordanian nurses' practice of PR is highly related to their level of knowledge (Nasrate et al., 2017). Thus, the poor levels of practice among nurses in this study might be attributed to the low level of knowledge and not merely their level of education. This implies the need for including PR subjects in the curricula of nursing education which can increase knowledge, change attitudes and potentially reduce the use of PR (Pellfolk et al., 2010).

### 5.1 Implications for nursing practice and policymaking

The findings of this study have implications for nursing practice. Most nurses did not verify patient identification before employing PR, did not introduce themselves to the patient and did not explain the procedures to the patient. Patients' rights, patients' safety and ethical and legal issues surrounding PR use must be emphasized through professional development programmes or special training to provide quality nursing care to patients.

Periodic assessment, observation and neurovascular checks must be emphasized through standard policies. Also, nurses should document their nursing care according to the documentation policy of the PR procedure. This highlights the need for clinical guidelines and educational programme on PR use in intensive care units. Clinical guidelines and educational programmes should clearly define the contraindications, documentation, time duration, indications, patient observation and the procedure itself. Further, it is essential to establish clear policies based on the best evidence and reviewed periodically to ensure safe practice.

Some hospitals have policies regarding PR, but their nurses are unaware of it. Administrators should disseminate the policies or standard practice guidelines regarding PR to their staff for consistent and safe practice. Adherence to policies must be encouraged through regular audit of patient charts or by direct observation of practice. In addition, the clinical environment can be improved through the provision of needed material and equipment for quality practice. The leaders of the ICUs were notified of the results of this study, and hopefully, they can start raising awareness about the existing policies on PR. Education and in‐service training can promote assessing the need, safe application and care related to PR.

Considering the patient's needs is very important when applying PR. Studies have shown that patients feel angry, helpless and hopeless when they are being restrained. Thus, there is a need to explore alternative methods of PR that could provide safety for patients without hurting them. Alternatives could be companionship, environment manipulation and sedatives (Azab & Negm, 2013). The observation was based on the competency checklist completed immediately after the PR application. The observers did not fill up the observation checklist in the presence of nurses.

### 5.2 Limitations

This study has some limitations. Nurses' knowledge was assessed using a self‐reported questionnaire and the observers filled up the observation checklist immediately after the PR application which might produce recall bias. Videotaping the procedure and analysing it later would have been more objective in validating the accuracy of the procedure. Also, not all PR applications were observed due to the lack of trained staff. Nevertheless, this study included observation methods that are more valid and more accurate. The convenience sampling method could affect the generalizability of the results. However, the study included hospitals from different sectors and included a large sample of nurses who are currently working in J ICUs in Jordan. The observers were coworkers, and we assumed collegial relationships. Closer friendships between the observer and observee might have existed and might have altered the validity of their observations.

## 6 CONCLUSION

PR use is a challengeable professional practice that nurses face in their duties. Nurses' lack of knowledge regarding PR use highlights the need for providing special education or training programmes for nurses in ICUs in Jordan to improve their knowledge which in turn could improve their practices regarding PR use. There is a need for building clear policies or standard guidelines related to PR use by the administrations of every hospital, and the hospitals should disseminate the related policies or standard guidelines to the nurses to enable them to provide safe care. Such changes could avoid litigations and provide safety for nurses and families.

## CONFLICT OF INTEREST

The authors have no conflicts of interest to declare.

## AUTHOR CONTRIBUTIONS

MA and WK and BQ: Concept and design of the study. BQ: Data collection and entry. MA and WK and BQ: Data analysis and interpretation of the results. MA, WK, BQ and RJ: Drafting and/or critically revising the manuscript. All authors read and approved the final version of the manuscript.

## Data Availability

Does your submission include any supporting information files for publication? No, this manuscript does not include any supporting information files for publication.
